# Case report: Immunotherapy guided by molecular profiling of tumors: illustrative cases and literature review

**DOI:** 10.3389/fmed.2024.1403056

**Published:** 2024-07-09

**Authors:** Mustafa Ozdogan, Eirini Papadopoulou, Vasiliki Metaxa-Mariatou, George Kapetsis, Angeliki Meintani, Chrysiida Florou-Chatzigiannidou, Akin Yildiz, Muharrem Okan Cakir, Onder Kirca, George Nasioulas

**Affiliations:** ^1^Clinic of Medical Oncology, Memorial Antalya Hospital, Antalya, Türkiye; ^2^Genekor Medical S.A., Athens, Greece; ^3^Department of Nuclear Medicine, Memorial Bahcelievler Hospital, Istanbul, Türkiye; ^4^School of Life Science, Pharmacy and Chemistry, Kingston University London, London, United Kingdom

**Keywords:** immunotherapy, TMB, NGS, molecular profiling, PD-L1, immune checkpoint inhibitor

## Abstract

Predictive biomarkers are necessary for the identification of immunotherapy-responsive patients. Tumor mutation burden (TMB), as determined by next-generation sequencing (NGS), and PD-L1 expression, as evaluated by Immunohistochemistry (IHC), are the biomarkers most frequently employed in clinical practice. In addition, microsatellite instability (MSI) was the first biomarker to demonstrate immunotherapy efficacy irrespective of the type of tumor and possesses a high predictive value. However, its limited use across most tumor types limits its therapeutic potential. This report describes two cancer patients with positive TMB and PD-L1 expression. The molecular profile of the tumor indicated that the first patient was responsive to Immune checkpoint inhibitors (ICI), while the second patient was resistant. These case studies demonstrate that tumor molecular analysis in combination with immunotherapy predictive biomarkers, such as PD-L1 expression and TMB, can enhance the prediction of response to ICI for specific patients. This methodology enables an individualized and improved approach to the treatment and management of the disease.

## Introduction

Immunotherapy with Immune checkpoint inhibitors (ICI) has significantly transformed the course of treatment for cancer patients. Diverse types of tumors have exhibited responses, and the number of approved ICI regimens continues to rise. Despite the efficacy of these drugs, several patients fail to show a response to the treatment, and they may experience severe adverse effects. Therefore, it is imperative to identify suitable predictive biomarkers of immunotherapy response to ensure the selection of patients who respond appropriately. Recently, the biomarkers that are most extensively utilized in clinical practice are PD-L1 expression, which is evaluated through immunohistochemistry (IHC), and tumor mutation burden (TMB), which is determined through next-generation sequencing (NGS) technology (1). In addition, microsatellite instability (MSI) has a high predictive value and was the first biomarker to demonstrate immunotherapy efficacy regardless of the type of tumor. However, its low incidence in the majority of tumor types limits its clinical use (2).

Even though these biomarkers have exhibited strong predictive values, it is still feasible to identify responders to treatment among biomarker-negative cases. In addition, positive PD-L1 and TMB levels do not ensure treatment response. In fact, their predictive value is poor in several tumor types. It is now indisputable that both the tumor characteristics and its microenvironment are substantial determinants of response (3). As a result, there is an increase in the significance of tumor molecular profile analysis, whereas specific somatic gene alterations appear to be predictive of immunotherapy treatment response. Therefore, the importance of tumor molecular profile analysis is becoming increasingly evident, while particular somatic gene alterations seem to guide immunotherapy treatment response prediction. As a result, the selection of treatment can be more effectively guided by the combined use of ICI biomarkers and the genetic background of the tumor. This provides additional information related to gene alterations affecting the tumor’s immunogenicity (4).

Two cases of cancer patients with positive TMB and PD-L1 expression, respectively, are presented in this report. In the first case, the molecular profile of the tumor suggested an ICI response, while in the second case, it suggested resistance. These cases provide evidence of the significance of tumor molecular analysis and the substantial support it can offer when combined with the results of immunotherapy predictive biomarkers, such as PD-L1 expression and TMB, to improve the accuracy of ICI response prediction in individual patients. This approach enables the development of a personalized and improved strategy for the treatment and management of the disease.

## Case 1 presentation

A 62-year-old male patient with reported abdominal pain and a carbohydrate antigen 19–9 (CA 19–9) value of 60.37 U/mL was subjected to the PET-CT scan, which revealed a hypermetabolic primary pancreatic malignancy with irregular contours in the pancreatic corpus. The malignancy extended to the superior and posterior positions, invading the splenic artery and vein, and with sporadic contact with the adrenal gland and stomach. In addition, the patient exhibited multiple abdominal tumor implants and pancreatitis. There was no report of smoking history, alcohol abuse, or a family history of cancer. Initial diagnosis was 5 days earlier on (01 August 2017), confirmed the presence of invasive ductal adenocarcinoma of the pancreas.

The initial treatment plan included palliative treatment weekly with modified FOLFIRINOX (mFOLFIRINOX) (5-fluorouracil, folinic acid, irinotecan, and oxaliplatin). The PET-CT scan after 8 weeks of mFOLFIRINOX showed partial response; however, signs of pancreatitis were almost completely resolved. The PET-CT scan was repeated after 16, 24, 32, 40, and 46 weeks with stable disease outcomes. In addition, molecular analysis was negative for MSI and PD-L1. After 46 weeks of mFOLFIRINOX treatment, the patient was forced to discontinue “oxaliplatin” due to toleration problems. Thereafter, the FOLFIRI treatment was continued until the 56th week of treatment when the PET-CT scan revealed an increase in the pancreatic tumor mass and peritoneal lesions.

To investigate the possibility of ICI implementation, a TMB analysis was recommended. The Oncomine Tumor Mutation Load Assay method (Thermo Fischer Scientific) was used, and it showed a high TMB value of 33.04 muts/Mb.

**Figure 1:**
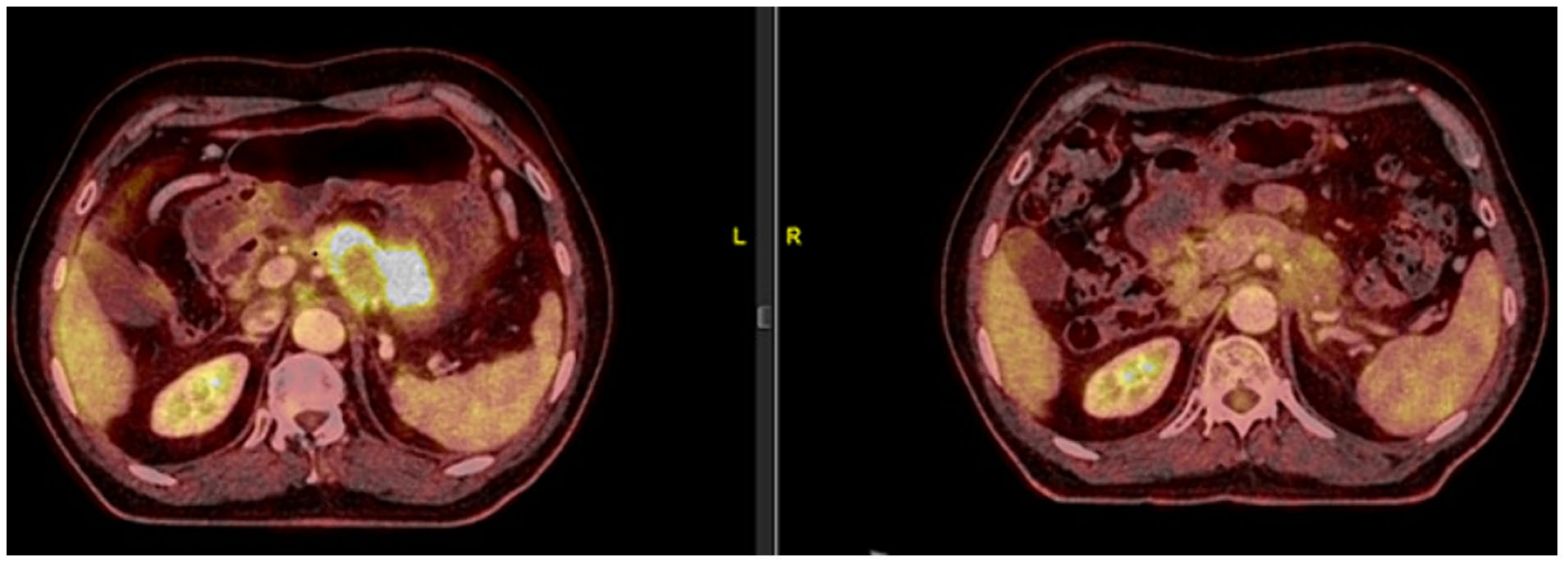
On 23 May 2019, the patient was started on 240 mg of nivolumab every 14 days. As the patient tolerated the treatment well, from the fourth cycle onwards, a 1 mg/kg dose of ipilimumab was added every 21 days. A significant partial response was observed in the PET-CT scan performed on 19 August 2019, after 6 cycles of nivolumab and 2 cycles of ipilimumab.

Based on this result, nivolumab was administered, almost 2 years after initial diagnosis (23 May 2019), with ipilimumab being added in the fourth cycle. The PET-CT scan, 3 months later, revealed a significant partial response (Figure 1).

In addition, since the patient had shown a good response to platinum based chemotherapy (oxaliplatin), a hereditary cancer gene panel test was used for investigating the hereditary variants conferring sensitivity to the enzyme poly ADP ribose polymerase (PARP) inhibitors such as Olaparib (5). However, no clinically significant variant was detected. Therefore, a somatic mutation analysis was conducted using the Oncomine Comprehensive Assay method (ThermoFisher Scientific) analyzing 161 unique cancer genes, including 87 oncogenes, 43 copy number amplification (CNA) genes, 48 tumor suppressor genes, and 51 fusion driver genes. The somatic inactivating alterations were identified in three genes, namely, the CDKN2B (cyclin-dependent kinase (CDK) inhibitor 2B), the FBXW7 (F-box and WD repeat domain containing 7), and the MLH1 (MutL Homolog 1) (Table 1). The first two genes are known as tumor suppression genes which are currently un-targetable, and there is evidence of their association with poor immunotherapy outcomes (6, 7). The MLH1 gene is a member of the mismatch repair (MMR) system. The variants in this gene are of particular interest since it has been shown that germline or somatic mutations can confer sensitivity to ICI treatment (8). Therefore, the high tumor mutation burden may be attributed to this mutation. The PET-CT scan showed a continuous response after 21 cycles of nivolumab.

**Table 1:** NGS analysis results using a multigene panel for tumor molecular profile analysis.

Biomarker	Result	VAF
*MLH1*	c.1292_1293insC (p.Met431fs)	16.7%
*FBXW7*	c.1629_1630delAG (p.Arg543fs) & c.1099C > T (p.Arg367Ter)	19.31% &13.44%
*CDKN2B*	c.244C > T (p.Arg82Ter)	40.52%

Immunotherapy was suspended for 2 months due to lung injury probably associated with the ICI treatment received, and viral infection with COVID-19 was diagnosed. Steroids were used as a treatment for autoimmune-mediated immunotherapy toxicities. Therefore, nivolumab treatment was continued, and the pancreatic mass and tumor implants were stable, showing a continuous response to the treatment. The patient received 96 cycles of nivolumab treatment up to 08 November 2023, with the disease remaining under complete control.

## Case 2 presentation

**Figure 2:**
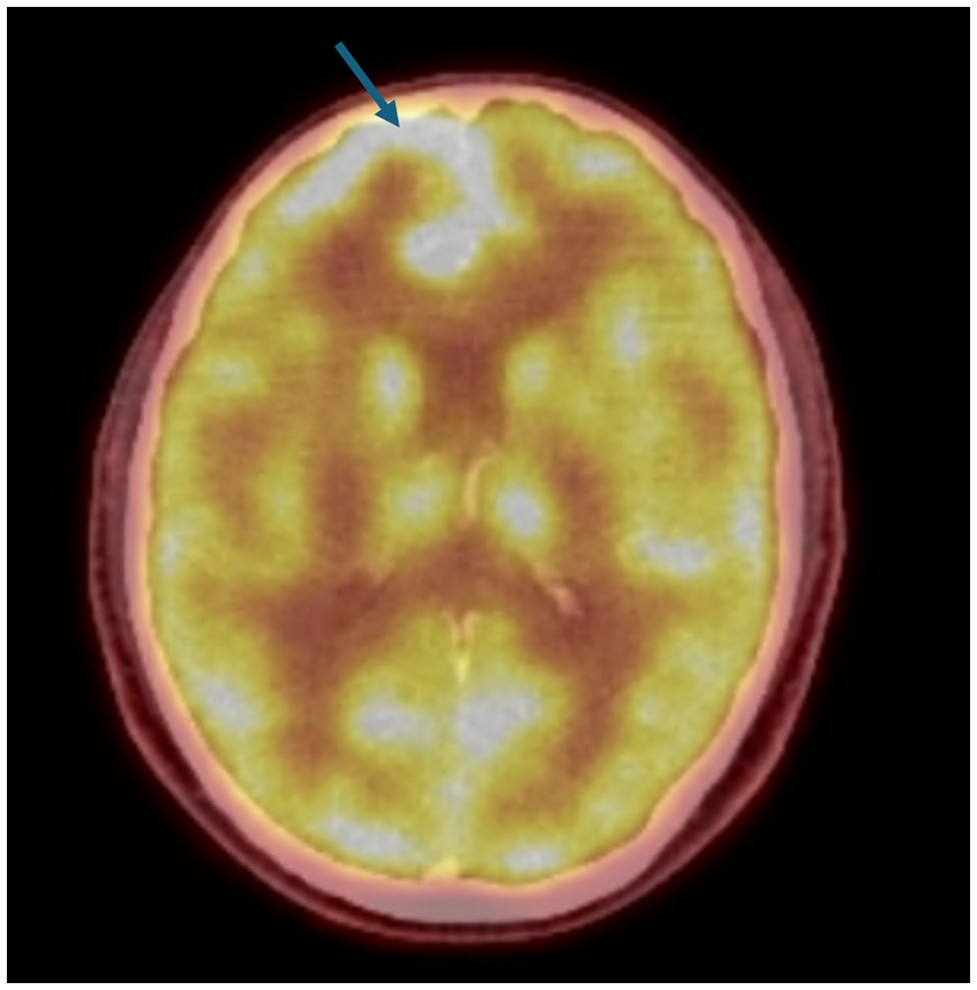
PET-CT: The PET-CT imaging dated 18 May 2023 indicates a suspicion of leptomeningeal disease characterized by diffuse, non-homogeneous hypermetabolic activity within the anterior, inferior, and medial regions of the right frontal lobe. This pattern of uptake is atypical and suggests a possible pathological process affecting the leptomeninges in these regions of the cerebral cortex, a novel finding as depicted in the figure provided.

A 51-year-old male patient with right upper lobectomy for early-stage lung adenocarcinoma (pT1b N0 M0) was diagnosed in 2018, with no adjuvant chemoradiotherapy conducted due to headaches in April 2023. The PET-CT scan on 18 May 2023 showed a new finding that was suspected to be leptomeningeal involvement due to diffuse, non-homogeneous hypermetabolism observed in the anterior, inferior, and medial regions of the right frontal lobe (Figure 2). No other metastatic lesion was detected. The Magnetic Resonance Imaging (MRI) spectroscopy conducted on 18 May 2023 primarily considered the lesion in the right frontal area at the parenchymal level as metastasis. However, the lumbar puncture (LP) test of cerebrospinal fluid (CSF) reported benign cytology.

Since there had been debates regarding the patient’s diagnosis of leptomeningeal metastasis and due to a history of adenocarcinoma biology, liquid biopsy analysis was considered. Therefore, the circulating tumor DNA (ctDNA) analysis of 50 cancer genes and 12 fusions was performed using the Oncomine Pan-Cancer assay (ThermoFisher Scientific) in both plasma and the CSF. There was no alteration identified in plasma ctDNA, while a KRAS p.G12S alteration was detected in the CSF, providing evidence of malignant metastasis. The initial treatment plan for isolated cranial metastatic disease included whole cranium radiotherapy that was completed on 02 June 2023. The cranial MRI scan did not show any difference from the previous scan conducted on 27 June 2023. Since no targetable mutations were detected, Carboplatin + Pemetrexed + Pembrolizumab treatment was planned. After 2 cycles, the PET/CT scan (August 14, 2023) showed decreased hypermetabolism. This was considered to be due to the previously observed leptomeningeal involvement around the right frontal lobe. The cranial MRI conducted on 11 August 2023 was consistent, and the disease was stable.

Meanwhile, comprehensive genomic testing was performed from the patient’s initial surgical tumor blocks of the 2018 lung adenocarcinoma. The analysis was performed using the oncology multi-gene variant assay method (GenePlus), a qualitative *in vitro* diagnostic test, that detects variants in 1,021 tumor-related genes and gene rearrangements/fusions in 38 genes. In addition, PD-L1 expression by IHC was performed. The results are summarized in Table 2.

**Table 2:** Tumor NGS/IHC analysis results.

Biomarker	Result	VAF	Approved therapies for indication	Therapies with potential benefit	Therapies with potential resistance
*KRAS*	Exon 2 c.34G > A (p.G12S)	8%	–	Cobimetinib (2C.1) Binimetinib (2C.1) Trametinib (2C.1)	–
*STK11*	Exon 4 c.492_493delinsTT (p.E165*)	10%	–	Everolimus (2C.1)	Pembrolizumab (2C.1) Nivolumab (2C.1) Atezolizumab (2C.1) Durvalumab (2C.1)
*MCL1*	Amplification	5.4 copies	–	–	–
*CDKN1B*	Amplification	5.4 copies	–	–	–
*DAXX*	Amplification	4.2 copies	–	–	–
*BCL2L1*	Amplification	4.2 copies	–	–	–
*NFKBIA*	Amplification	4.2 copies	–	–	–
*B2M*	Amplification	4 copies	–	–	–
Microsatellite instability (MSI)	Stable (MSS)		–	–	–
Loss of heterozygosity (LOH)	Low 7.43%		–	–	–
PD-L1 Expression	Positive TPS > 50%		Pembrolizumab (1A.1) Nivolumab (1A.1) Atezolizumab (1A.1) Durvalumab (1A.1) Nivolumab+Ipilimumab (1A.1)	–	–
Tumor mutational burden (TMB)	7.68 muts/Mb		–	–	–

Variants’ Level of Evidence (LoE) (e.g., 1A.1, 2C.1, and 1B) are based on the Joint consensus recommendation of AMP, ACMG, ASCO, and CAP for reporting genetic variants in cancer (4). VAF: Variant allele frequency.

**Figure 3:**
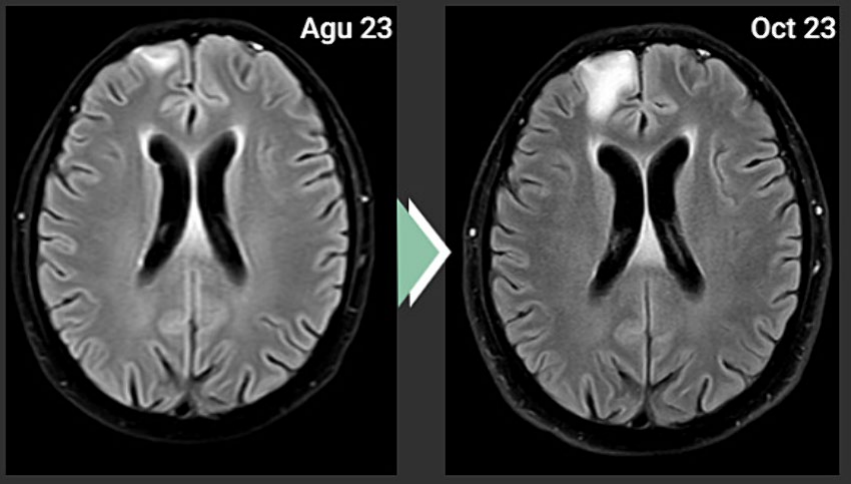
Cranial MRI showing progression.

Based on the positive PD-L1 value, an off-label administration of Carboplatin + Pemetrexed + Pembrolizumab was decided. However, the cranial MRI scan after 4 cycles of this treatment showed progression (Figure 3). There was no metastatic lesion detected in the PET-CT scan for restaging purposes, other than the progressive lesion in the brain. As a second line of systemic therapy, Paclitaxel + Gemcitabine was started.

The key tumor alteration driving primary resistance to immunotherapy seems to be the STK11 inactivating variant, which is present in the primary adenocarcinoma. The STK11 alterations with a cold tumor immune microenvironment and are characterized by a low level or absence of PD-L1 biomarker. They have been identified as an important regulator of resistance to anti-PD-1/PD-L1 therapies and poor outcomes with chemoimmunotherapy (9, 10).

## Discussion

The use of predictive biomarkers for ICI treatment is essential for appropriate patient selection.

PD-L1 expression, assessed using Immunohistochemistry, is the most widely used ICI biomarker. However, it is also well known that this is not an ideal biomarker, and it is not the sole predictor of response to checkpoint inhibition.

Tumor mutation burden (TMB) refers to the number of somatic mutations in the coding region, usually indicated as the total number of somatic mutations within each MB of the tumor genome region. The clinical utility of TMB as a predictive biomarker for anti-PD1 immunotherapy has been established in the KEYNOTE-158 trial which led to the site-agnostic FDA-approval of pembrolizumab for metastatic/untreatable solid tumors with tissue TMB value of ≥10muts/Mb (11).

If either PD-L1 expression or TMB is positive, the likelihood of a response is increased; however, the maximum benefit from ICI selection appears to be achieved when both are positive. This is because a higher level of PD-L1 expression suggests a greater likelihood of an immunotherapy response, whereas a high TMB level can result in the production of neoantigens that can elicit an immune response (12).

Furthermore, the landscape of ICI response prediction is evolving to incorporate a more comprehensive understanding of the tumor microenvironment and immune interactions, as research continues to uncover new biomarkers and refine the existing ones. The combination of emergent biomarkers in clinical practice enables healthcare providers to more effectively identify the patients who are likely to benefit from immune checkpoint inhibitors and optimize treatment strategies for improved outcomes in cancer patients.

The tumor-infiltrating lymphocytes (TILs), gut microbiome composition, gene expression profiles, biomarkers related to tumor metabolism, and gene expression profiling are among the additional biomarkers that are emerging for ICI response prediction (13).

The alterations in specific genes and molecular pathways within the tumors can also have a significant impact on the response to immune checkpoint inhibitors in cancer immunotherapy. These tumor alterations can influence the tumor microenvironment, immune response, and overall sensitivity to immune checkpoint blockade. The genetic alterations in oncogenic pathways, such as mutations in genes like *EGFR, KRAS, and BRAF*, can impact the immune response in the tumor microenvironment (13, 14). The activation of certain oncogenic pathways may promote immune evasion and resistance to immunotherapy, while inhibition of these pathways may enhance response to ICI (15).

Therefore, it is crucial to comprehend the tumor alterations that can influence the response to ICI treatment to enhance the outcomes of cancer patients and optimize treatment strategies. Thus, a comprehensive genomic profile by NGS is frequently implemented in metastatic cancer patients.

In the first case presented, the analysis of an established ICI biomarker, TMB, showed evidence of ICI treatment responsiveness. In addition, a multi-gene tumor NGS analysis revealed the presence of a monoallelic MLH1 variation. This, in addition to the observed elevated TMB value, provides additional evidence of ICI effectiveness in this patient.

The TMB-high pancreatic cancer patients usually belong to a specific subset of patients with prolonged survival harboring further actionable alterations and displaying strong anti-tumor cytotoxic T-cell-mediated immune response (16). The presence of a monoallelic *MLH1* alteration may not lead to microsatellite instability; rather, it may be a random variation caused by the tumor’s propensity to acquire alterations, which consequently increases the burden of neoantigens (17).

*The MLH1* gene belongs to the mismatch repair pathway (MMR), which is one of the primary DNA repair mechanisms. It is used by the cells to correct base–base mismatches induced by the erroneous incorporation of nucleotides during DNA synthesis. The germline inactivating variants in the primary MMR genes (*MSH2, MLH1, MSH6, PMS2,* and *EPCAM*) have been associated with the hereditary cancer syndrome, the Lynch syndrome (LS). Recently, the impact of the somatic inactivation of such genes has been studied, revealing an increased tendency of mutation accumulation and probable susceptibility to ICI treatment (18).

Therefore, nivolumab treatment was a viable option for patients with such biomarkers and had proven effective in this patient.

In the second case, liquid biopsy analysis in the CSF was used to confirm the diagnosis of leptomeningeal metastasis, which is difficult to diagnose by the major imaging and pathological evaluation. Furthermore, despite the positivity in the PD-L1 expression indicative of ICI response, the patient progressed upon treatment administration. Brain metastases are generally assumed to have a cold tumor immune microenvironment and are predicted as poor responders to ICI treatments. However, several studies have also shown the benefit of pembrolizumab administration in these patients (19, 20). Pembrolizumab has demonstrated efficacy in treating a variety of cancer types, including melanoma and non-small cell lung cancer, and there have been promising results in patients with brain metastases (19, 21). In addition, ongoing research is focused on exploring ways to improve the response of brain metastases to ICIs, such as combining treatment with other therapies or optimizing dosing regimens (19, 22). Therefore, an off-label combination treatment of Carboplatin+Pemetrexed+Pembrolizumab was implemented.

However, when the tumor molecular profile of the patient was made available, an *STK11* alteration was detected, indicating that resistance to treatment was highly likely in this case. Mutations in the serine/threonine kinase 11 gene (*STK11*), also known as liver kinase B1 (*LKB1*), are detected in approximately 5–30% of NSCLC cases (23). The *STK11* alterations are associated with a cold tumor immune microenvironment characterized by low or no PD-L1. They have been identified as an important regulator of resistance to anti-PD-1/PD-L1 therapies and with poor outcomes in chemoimmunotherapy (9, 10). The prognostic role of *STK11* mutations in combination with co-occurring alterations in other cancer genes is being evaluated in various studies, with poor ICI outcomes observed in patients with low TMB or *KRAS* co-mutation (24).

Both these cases indicate that TMB and PDL-1 provide only a piece of the information regarding ICI response prediction, whereas, the tumor alterations detected by NGS analysis could strengthen or weaken the probability of treatment effectiveness. The tumor test can provide information for the selection of both appropriate targeted treatment and immunotherapy. Therefore, a thorough evaluation of biomarkers for both ICI and targeted treatment increases the number of viable treatment options. This could assist in better comprehension of the tumor’s biology, which is crucial in deciding whether immunotherapy is a suitable approach in each case, or an alternative treatment option should be evaluated.

## Data availability statement

Data and materials supporting the conclusions of this article are available upon request to the corresponding author.

## Author contributions

MO: Conceptualization, Writing – original draft. EP: Data curation, Methodology, Writing – original draft. VM-M: Methodology, Writing – review & editing. GK: Methodology, Writing – review & editing. AM: Data curation, Writing – review & editing. CF: Methodology, Writing – review & editing. AY: Methodology, Writing – review & editing. MC: Data curation, Writing – review & editing. OK: Data curation, Writing – review & editing. GN: Conceptualization, Writing – review & editing.

## Ethics statement

The studies involving humans were approved by Clinic of Medical Oncology, Memorial Antalya Hospital. The studies were conducted in accordance with the local legislation and institutional requirements. The participants provided their written informed consent to participate in this study. Written informed consent was obtained from the individual (s) for the publication of any potentially identifiable images or data included in this article.
